# Beyond the Bench: Taking Action in Northern Manhattan

**Published:** 2005-02

**Authors:** Swati Prakash

Residents of Northern Manhattan face exposure to a multitude of environmental hazards, ranging from those within the home (such as lead-based paint, pesticides, pest allergens, and mold) to a myriad of neighborhood exposures (including two sewage treatment plants and six out of seven of Manhattan’s diesel bus depots). Communities of Northern Manhattan also bear a disproportionate share of adverse health outcomes—including coronary heart disease and childhood asthma hospitalizations—that are potentially linked to these and other environmental exposures. Residents of Northern Manhattan have a rich history of organizing to promote health and challenge toxic environmental exposures, but have often lacked access to the technical and informational resources to help them understand and prioritize health risks. Now that roadblock is being dismantled.

In 1997, community environmental health focus groups at three Northern Manhattan sites identified air pollution, garbage, water quality, asthma, exhaust from heavy traffic, indoor air quality, and quality-of-life issues as the most pressing environmental concerns for those communities. Based on these responses, the Community Outreach and Education Program (COEP) of Columbia University’s NIEHS Center for Environmental Health in Northern Manhattan, in collaboration with community partners West Harlem Environmental Action and the Harlem Center for Health Promotion and Disease Prevention, developed a program known as Environmental Health Leadership Training (EHLT).

The goal of the EHLT is for community residents of Northern Manhattan and the South Bronx to improve their capacity to organize for community environmental health and justice in New York City. They do this by learning the scientific and regulatory foundation of environmental health issues affecting New Yorkers, as well as by learning basic organizing and advocacy skills to address health disparities in environmentally influenced health outcomes.

Four rounds of this training, which has been expanded to a 24-credit-hour curriculum, were held between 1997 and 2004. The structure for each session was a combination of small group activities and lectures by Swati Prakash, COEP coordinator and the central trainer for the EHLT, and guest lecturers, primarily researchers from the Columbia Mailman School of Public Health. The training is designed to convey sophisticated technical and health information while remaining accessible to people from a variety of educational backgrounds, literacy levels, and ages. Upon graduation, leaders are given a 200-page manual with extensive written and visual resources on all the issues addressed in the training.

To date, 85 community leaders have graduated from the training. Many EHLT graduates have gone on to play significant roles in setting public health policy at the citywide, statewide, and national level. EHLT participants testified in 1999 at the U.S. Environmental Protection Agency’s public hearings on its draft heavy-duty diesel engines rule to reduce emissions of asthma-exacerbating diesel exhaust. The presence of EHLT graduates was, in fact, the sole counterbalance to heavy industry presence at the New York hearings. Several graduates have also become key leaders in a successful campaign to pass city legislation to better protect children from lead-based paint hazards in the home. Graduates of the most recent training are currently preparing to launch a Healthy Homes campaign to address endemic problems of mold and pesticide exposures in Northern Manhattan housing.

The EHLT will continue with a citywide workshop in April. The COEP and its partners also hope to make the training available to other organizations by next year.

## Figures and Tables

**Figure:**
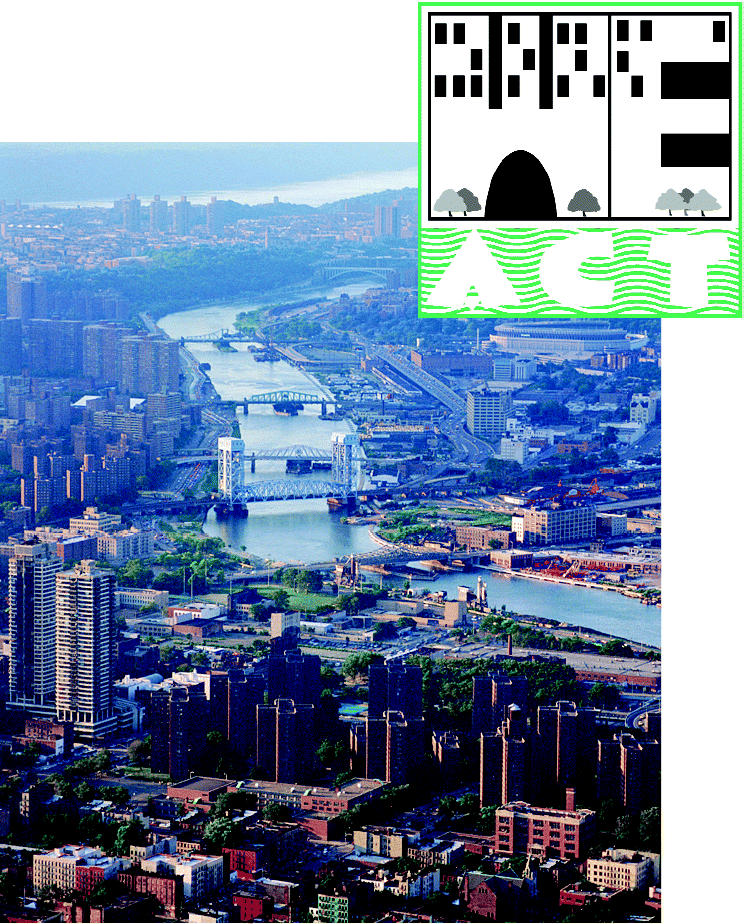
**Leaders in residence.** COEP training helps build community leaders who can take charge on environmental issues.

